# Factors influencing ethnic minority medical students’ performance in second sit examination

**DOI:** 10.3389/fmed.2026.1870616

**Published:** 2026-08-05

**Authors:** Olanrewaju Sorinola, Rebecca Mole, Emily Unwin, Laura Cranshaw, Laura Wopara, Casie Osborne, Collette Clay

**Affiliations:** 1 Warwick Medical School, University of Warwick, Coventry, United Kingdom; 2 Warwick Business School, University of Warwick, Coventry, United Kingdom; 3 Politics and International Studies (PAIS) Department, University of Warwick, Coventry, United Kingdom

**Keywords:** awarding gap, ethnic minority, examinations, UK, undergraduate medicine

## Abstract

The variation in levels of performance on educational assessments between students from different societal groups where ethnic minority (EM) students are awarded lower academic outcomes compared to their white counterparts remains a persistent issue across all UK higher education. At Warwick Medical School, the largest graduate entry medical school in the UK, a detailed analysis of all examinations from 2015 to 2024, across all three phases of the medicine course, showed that a statistically significant higher number of EM students end up in second sit examinations in comparison with their white peers. However, in the second sit examinations there was no statistically significant difference in performance. Therefore, the research aim was to explore factors that facilitated EM students’ improved performance in second sit examinations and if those factors could be transferred to first sit examinations, the outcomes of which are used for honours degree award. We used a qualitative design and interviewed medical students that have failed, resat their examinations and passed. Thematic analysis revealed four key themes: socio-cultural factors, resources, support and personal factors that influenced performance and how addressing these factors improved students performance. The study highlighted the complex and multifaceted factors that contributed to failure and identified strategies to improve performance.

## Introduction

The awarding gap is defined as the difference in proportion of white versus ethnic minority (EM) students awarded an undergraduate 1st or 2nd class upper degrees (1). Differences in degree attainment in higher education between various ethnic groups were reported in the 1990s (2) and further studies have subsequently confirmed these differential attainments (3, 4). This variation in levels of performance on educational assessments between students from different societal groups (5) where EM students are awarded lower academic outcomes compared to their white counterparts, though now well-documented remains a persistent issue across all UK higher education (6–8). It has therefore become an area of focus for sector policy (Office for Students in the UK) and consequently institutional strategies. The problem is not limited to UK higher education though, as research from other parts of the world has demonstrated the same degree award disparity (9). Existing literature has been useful in establishing the problem, identifying patterns in awarding gap data and highlighting the systemic nature of the inequality. Some authors have focused on the role of policy and assessment type in narrowing the awarding gap (7), while others have argued that much of the existing research does not adequately capture the nuanced, lived experiences of EM students (6, 8).

While the definition and measurement of the awarding gap is clear with undergraduate 1st and 2nd class upper degree award differentiation, the nature of medical degree awards as pass or pass with honours makes it more nuanced and challenging to identify, define and demonstrate an awarding gap in medical education. The prevailing approach is to measure awarding gap as the difference in the proportion of white versus ethnic minority students achieving honours in their medical degrees. Based on this, various authors have explored the reasons for the awarding gap in medical education. Woolf et al. (10) showed that ethnic group had an independent and negative effect on examination performance, and although ethnic differences existed on a number of demographic variables, the relationships between ethnic group and examination performance was found to be unmediated by age, socio-economic group, sex, schooling, parents’ education, language, personality, study habits, or motivation. Similarly, Jones et al. (11) and Brown et al. (12) have demonstrated the ethnicity awarding gap in medical education and the influence of ethnicity on the awarding gap. The studies have suggested that further exploration is needed with regards to the specific, contextual factors that contribute to awarding gap disparity in medical schools and the need to understand EM students’ experiences.

**Figure 1:**
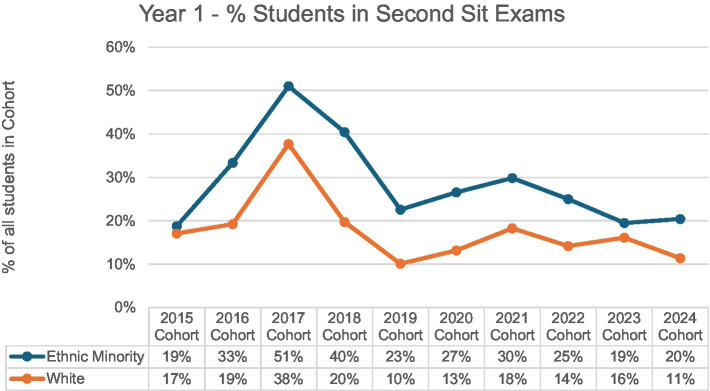
Phase 1 examination outcomes by ethnicity.

**Figure 2:**
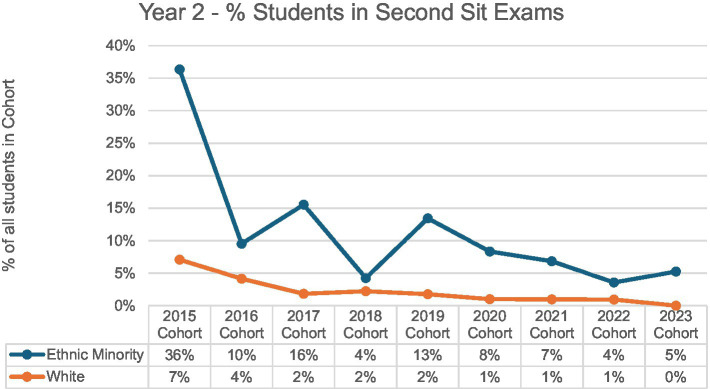
Phase II examination outcome by ethnicity.

**Figure 3:**
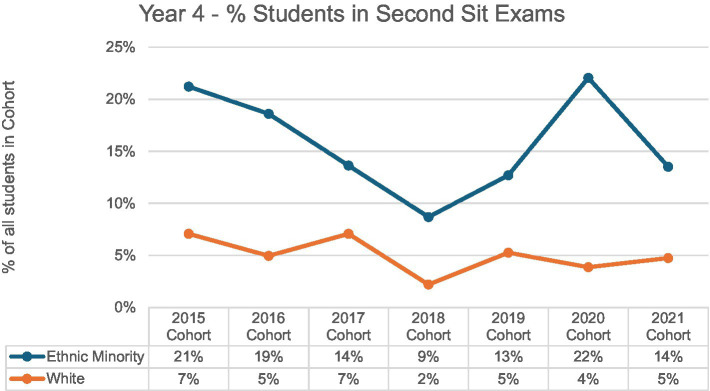
Phase III examination outcome by ethnicity.

Warwick Medical School (WMS) is the largest graduate entry medical school in the UK with students needing a 1st or 2nd class upper degree in their first course for admission to study medicine. Despite this prior high achievement, at WMS we have established in previous studies the existence of an awarding gap between our white and EM students and explored EM student experiences that contributed to this differential attainment (13–15). Further detailed analysis of all our examinations from 2015 to 2024, across all three phases of the medicine course, showed that a higher number of EM students (range 10–25%) end up in second sit examinations in comparison with their white peers and this was statistically significant (Figures 1–3, Table 1). This analysis by Phase, i.e., Phase I (Year 1), Phase II (Year 2) and Phase III (Years 3 and 4—no examination in Year 3), further clarified that the significant difference in performance was irrespective of the assessment type being written knowledge focused or clinical knowledge focused. However, in the second sit examinations over the same time period, there was no statistically significant difference in performance between EM and white students highlighting a narrowing of the performance gap between EM and white students in the second sit examinations (Table 2).

**Table 1:** Combined MBChB first sit examination outcomes by phase and ethnicity (2015–2024).

Phase	Ethnicity	Outcome			Chi^2^ and *p* values
		Passed first sit	Failed first sit	Total	
Phase 1	Ethnic minority	455 (74.96%)	152 (25.04%)	607 (100%)	Chi^2^ = 23.35 *p* < 0.0001
	White	995 (84.39%)	184 (15.61%)	1,179 (100%)	
Phase 2	Ethnic minority	449 (89.98%)	50 (10.02%)	499 (100%)	Chi^2^ = 43.16 p < 0.0001
	White	982 (97.71%)	23 (2.29%)	1,005 (100%)	
Phase 3	Ethnic minority	321 (86.52%)	50 (13.48%)	371 (100%)	Chi^2^ = 29.24 *p* < 0.001
	White	768 (95.40%)	37 (4.60%)	805 (100%)	
All phases	Ethnic minority	1,225 (82.94%)	252 (17.06%)	1,477 (100%)	Chi^2^ = 79.28 p < 0.001
	White	2,745 (91.84%)	244 (8.16%)	2,989 (100%)	

**Table 2:** Combined MBChB second sit examination outcomes by phase and ethnicity.

Phase	Ethnicity	Outcome			Chi^2^ and *p* values
		Passed second sit	Failed second sit	Total	
Phase 1	Ethnic minority	117 (76.97%)	35 (23.03%)	152 (100%)	Chi^2^ = 1.1886 *p* = 0.276
	White	132 (71.74%)	52 (28.26%)	184 (100%)	
Phase 2	Ethnic minority	45 (90.0%)	5 (10.0%)	50 (100%)	Fisher’s exact (2-sided) *p* > 0.05
	White	21 (91.3%)	2 (8.7%)	23 (100%)	
Phase 3	Ethnic minority	41 (82.0%)	9 (18.0%)	50 (100%)	Chi^2^ = 1.7499 *p* = 0.186
	White	34 (91.89%)	3 (8.11%)	37 (100%)	
All phases	Ethnic minority	203 (80.56%)	49 (19.44%)	252 (100%)	Chi^2^ = 1.131 *p* = 0.287
	White	187 (76.64%)	57 (23.26%)	244 (100%)	

Therefore, this research project aimed to explore factors that contributed to EM students’ improved performance in second sit examinations and whether they could be transferred to first sit examinations, the outcomes of which are used for honours degree award. While it does not seek to fully explain why the awarding gap exists, it provides valuable insight that could inform strategies to improve EM students’ examination performance. Our research question was: What factors facilitate EM students’ improved performance in second sit examinations?

## Methodology

To answer this research question, we adopted an exploratory qualitative design using a phenomenological approach (16, 17). This methodology was chosen to capture rich, in-depth insights into understanding the lived experiences and challenges EM students face in relation to medical school examinations which could not be obtained through quantitative methods alone. Situated within this paradigm, we adopted a constructivist framework so that participants’ lived experiences and meaning-making practices contribute to the co-construction of findings. This ensured that knowledge and understanding was generated through interaction, dialogue and dynamic relationship between researchers and participants (18). The COREQ (COnsolidated criteria for REporting Qualitative research) standards informed our study design, data analysis and reporting.

Participant recruitment for the study was voluntary but focused on WMS medical students who had failed at least one examination and had to resit the examination. A total of 12 participants were recruited, representing a range of student characteristics including male, female, commuter status, caring responsibilities, and neurodiversity. The recruitment process was carefully aimed and designed to capture diverse EM student voices and experiences. The ethnicity categories of the student participants are shown in Table 3.

**Table 3:** Study participants ethnicity categories.

Ethnicity	Number of students
Asian or Asian British	
Asian—Indian	3
Asian—Pakistani	1
Asian—Bangladeshi	2
Any other Asian Background	1
Black—African or African British	
Black African	3
Black Caribbean	1
Any other Black background	1

Data was collected through semi-structured interviews, conducted as both individual sessions and focus group sessions: four individual interviews and two focus groups were conducted. All sessions were conducted online via Microsoft Teams to accommodate students’ busy schedules. Interviews were conducted by two members of the research team (LW and CO) who were experienced at conducting qualitative interviews. Each session lasted about an hour, was audio-recorded, and transcribed verbatim, with transcripts double-checked by participants for accuracy. Using both interviews and focus groups enabled the capture of complementary forms of qualitative data. The interviews enabled in-depth explorations of participants’ experiences and perspectives, while the focus groups surfaced shared understandings and points of consensus and divergence to emerge. Using both approaches enabled the authors to capture both individual experiences and group dynamics. It also enabled participants to contribute in ways that best suited their communication preferences—enhancing inclusivity.

The semi-structured topic guide had open-ended questions to allow participants’ broad interpretation and to share experiences in their own words. The interview guide was organised around three main areas: participants’ perspectives on reasons behind examination failure, their perspectives on what facilitated success in the second sit examination and exploration of potential solutions to close the differential attainment gap. This structure focused on providing a rich understanding of the factors influencing examination outcomes, ensured that discussions captured challenges and successes while exploring potential solutions from the students’ perspectives. This approach also enabled participants to raise issues not anticipated by the research team and ensured that the findings reflected participants’ perspectives rather than imposing pre-determined categories or assumptions. Interviews were conducted until no new themes emerged from the data and thematic saturation had been achieved.

Ethical approval was obtained from the University of Warwick ethics research committee. Written informed consent, agreement for audio recording and use of transcripts was obtained from all participants. All data were anonymised and securely stored in accordance with Warwick University data protection policies.

## Data analysis

The data collected were analysed using Braun and Clarke’s (19) six-step framework for thematic analysis, allowing for the identification of key themes emerging directly from the participants’ narratives. This approach allowed the study to combine deductive coding (based on research questions) with inductive coding to capture themes that emerged directly from participant narratives. The coding and organisation of data were undertaken using N-Vivo which facilitated sharing and collaboration among the research team. All research team members read and familiarised themselves with all transcripts to gain an overall understanding of the data and identify initial ideas. Each transcript section was then independently coded and emerging codes were used to identify preliminary themes. Each section was reviewed by at least two researchers to minimise personal bias and enhance reliability during the initial coding process. During this process, the research team met regularly to discuss codes and preliminary themes and work collaboratively to establish consensus. Through this iterative process, codes were refined and organised into a final thematic framework to best reflect participants’ perspectives and experiences. This systematic approach provided a rigorous and transparent method for identifying patterns in the data, allowing the study to generate rich, in-depth insights into the factors influencing examination outcomes among EM students.

### Reflexivity

Five of the authors (OS, RM, EU, LC & CC) are based at WMS and involved in the delivery of the MBChB programme, and OS, RM and CC are also involved in the assessment process. All five authors are members of the WMS Awarding Gap Group that actively explores causes of the awarding gap. While the authors acknowledge that this could be a source of bias, however, to mitigate this, during the conceptual design, we deliberately had the post graduate students LW and CO conduct the interviews and focus groups. Even though based at UoW, LW and CO are not involved with WMS or the assessment process thereby reducing the risk of assumptions and bias at the data collection phase. The authors’ ethnicities are also different with two Blacks and five Whites ethnic distribution. Furthermore, to minimise bias, multiple coders coded the data to capture multiple perspectives, and the authors had regular team discussions throughout the research process.

## Results

**Figure 4:**
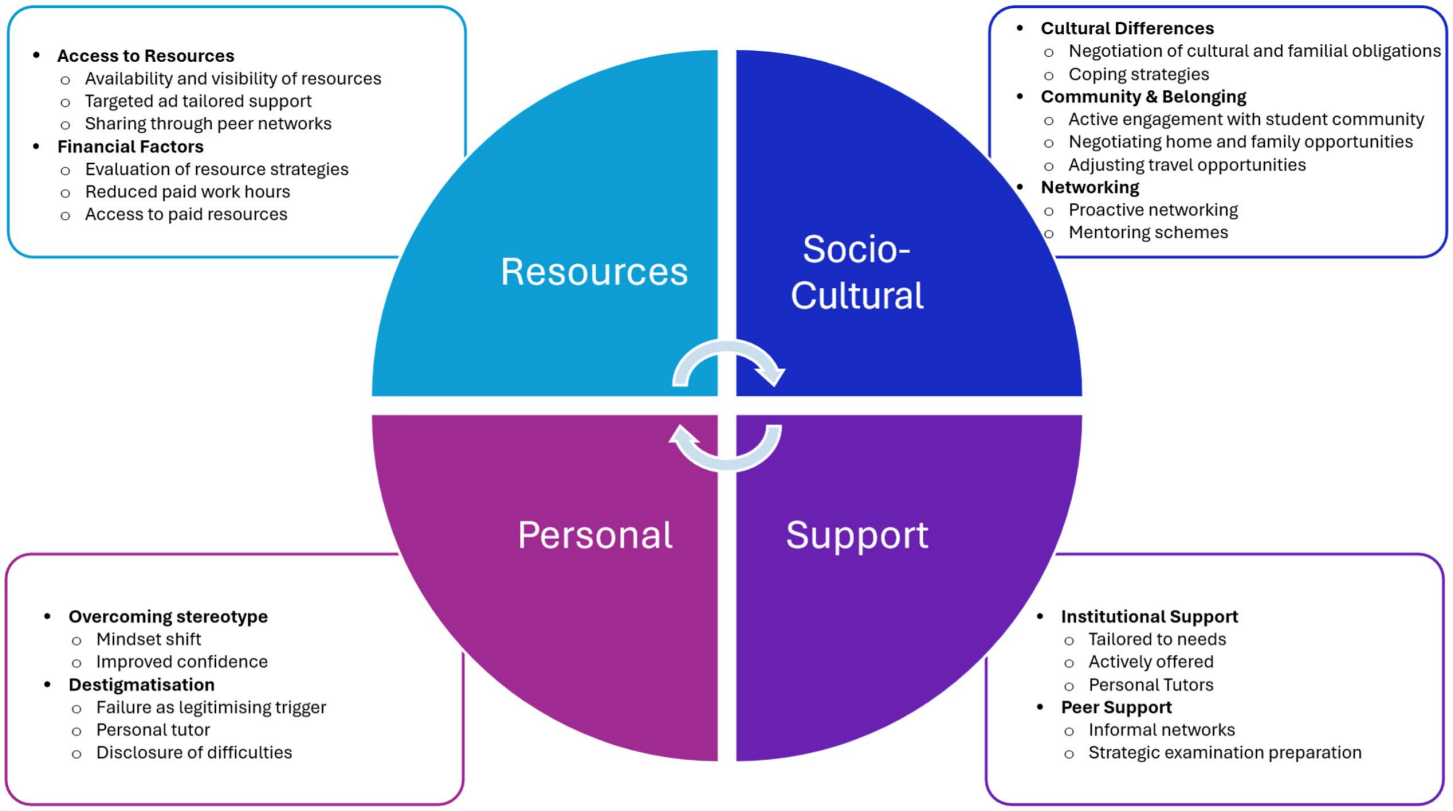
Themes and subthemes identified as contributing to success in second sit examinations.

Four key themes and their corresponding subthemes were identified from the interviews and focus groups with medical students (Figure 4). These themes described the main factors that students attributed their failure at the first sit of examinations to be due to, and the factors they actively addressed which they perceived helped to improve their performance at the second sit examinations.

### Theme 1: socio-cultural

Socio-cultural factors emerged as a very strong theme describing the social and cultural dynamics that influenced participants’ academic experiences when preparing for examinations. It revealed how the dynamics of engagement with learning by students from ethnic minority backgrounds is highly influenced by cultural differences, sense of community and belonging, networking practices and the impact all these had on their ability to succeed at examinations.

#### Cultural differences

Participants described how cultural and family obligations competed directly with academic demands and how the conflicts became a burden for them. Participants described a significant “dual burden” of pressure which they felt was distinct from their white peers. This was described as high parental expectations to succeed (e.g., “work two times harder”) and time-consuming family or cultural responsibilities (such as marriage obligations, managing household issues, etc.). Participants emphasised the need to honour their own heritage, respect cultural backgrounds and overcome perceived stereotypes but at the same time reflected that these cultural differences with family commitments, expectations and at times lack of family support significantly shaped and contributed to their failure in examinations.

*“For me, university is kind of a little selfish thing that I get to indulge in… especially in my culture as a girl it is optional to go to school as long as it is not interfering with my husband’s work.”*

*“For example, I’m married, and if my in-laws had a social event, even if I don’t think it’s very important, I would still have to go. I think a lot of my peers who are white tend to move out to university, and then that is kind of their world.”*

*“A lot of people I’ve met on the course who are from ethnic minority backgrounds tend to have culturally more responsibilities back home that’s of like a higher significance than their own studies.”*

There were also reports of the burden of cultural taxation and the additional workload placed on students from EM groups to educate their peers about their group simply because of their identity, and the impact of this on their own learning and sense of belonging.

*“In CBL… there was a problem with Black maternal death rate, and I was the only Black girl in my group. They were looking at me to explain everything… I’m here to learn properly; I shouldn’t be the one in charge of teaching.”*

To negate the challenges perceived to be caused by cultural differences, some participants purposefully renegotiated with their families to rebalance learning priorities by renegotiating home and family priorities, meeting less family expectations and prioritising attendance at study sessions to achieve examination success. Participants also described how developing effective coping strategies to manage the emotional impact of cultural pressure, subsequently led to increased self-confidence and in turn they perceived this to influence their chance of success in second sit examinations.

#### Community and belonging

Participants recounted a sense of disconnection and isolation from the medical school community and the wider university. Participants recognised that their sense of belonging, being valued within a community of learners, and engaging in networks was influenced by their perceived cultural differences. They felt being an EM and underrepresented was an issue leading to lack of integration and exclusion from study groups.

*“I was also at home for all that time… I didn’t really get a chance to integrate with other students or have a chance to interact and study together with other people.”*

Furthermore, EM students reported that this lack of community and sense of belonging was exacerbated by social activities that were perceived to be non-inclusive with limited opportunities for meaningful peer interaction. This impacted their ability to foster and develop friendships.

*“I was acutely aware that I did not really have friends, whereas everyone else was bonding a lot more outside of timetabled sessions… that makes a big difference because what I didn’t realise, is everyone is practising OSCEs just in their friendship groups or like if someone learns something, you hear about what that person’s learnt about.”*

As participants started to prepare for their second sit examinations they described how they actively pursued opportunities to enhance their engagement within the student community by forming focused study groups and practicing with other students (both white and EM groups) which provided structure and reassurance.

*“So, to prepare for the second sit, it was just practice, practice, practice in person, practice with peers who had passed from all different groups…”*

For others, sense of belonging was rebuilt through shared experiences with fellow resitters, which reduced feelings of isolation / loneliness, increased motivation and facilitated engagement with collaborative learning, contributing to improved resit performance. Other students described making practical changes such as spending more time on campus, relocating closer to school and peers. So there was a definite shift in students’ sense of belonging within the medical school community. Whereas feelings of isolation and disengagement characterised their first-sit experience, failure prompted more deliberate efforts to build connections, purposeful interactions and integration both academically and socially resulting in an improved sense of belonging and being part of a community.

#### Networking

Participants recognised that networking and examination success were intertwined. They reported missing out on informal learning opportunities and access to additional learning resources by not belonging to the community of learners.

*“I did without realising, miss out on a lot of education because there’s a lot that happens informally and that’s what actually makes the difference.”*

*“I didn’t really have a good peer support network…. I could see a lot of the other students (White) had formed this peer support network. They were all kind of motivating each other… I didn’t have that”*

Failure appeared to act as a catalyst for seeking out information that had previously been inaccessible or unknown. Students became more proactive in joining group chats, seeking advice from peers who had passed examinations and engaging with students in upper years. Peer networks were described as central to the informal sharing of resources and strategies and gaining access to the informal curriculum.

*“Group chats on WhatsApp…unless your’e not part of that group, you will feel a little bit lost.”*

*“Group chats. I think having an insight into how others are doing in their revision, and a lot of EM students aren’t aware of these group chats they can access.”*

Participants reported that accessing the peer group chats improved their focus, reduced self-doubt and prepared them not only for the second sit, but enabled them to gain new skills to prepare them for future examinations.

*“I’d say the two main changes (for success) were probably more recall and… a more supportive, specifically, peer study network.”*

### Theme 2: resources

Resource-related constraints emerged as key influential determinants of differential performance between first sit and second sit examinations. Across accounts, participants emphasised that it was not simply the presence of resources, but the availability, visibility, accessibility, and timing of academic resources that shaped their preparedness. Limited awareness of key materials, inconsistent access pathways, and structural barriers impeded first sit performance, whereas clearer, more targeted and expanded access following failure was perceived to facilitate improved outcomes in the second sit examination.

#### Access to resources

Participants described differences between access and availability of resources before the first sit and those encountered prior to the second sit examinations. Prior to the first sit examination, many participants reported limited clarity about which learning materials existed, how assessments would be marked, or what constituted effective examination preparation. Participants described their poor and inconsistent awareness of resources encompassing both formal university-provided resources (such as mark schemes, question banks, exam-style answers, structured revision tools) and informal resources such as peer-shared materials and group chats.

*“I think that there were resources that perhaps I wasn’t aware of or I kind of didn’t have access to prior to that* [first sit examination]*.”*

Following failure of the first sit, participants described a marked shift in the availability and visibility of resources. Participants reported that second sit students received additional tailored guidance from staff, targeted revision sessions, and informal sharing of resources through peers which made key materials more accessible. This transition enabled more efficient, focused, and strategic study behaviors.

*“I would message the second-year girl that I was paired up with… and then she sent me resources and then she’d be like ‘OK, these resources are from here’”*

*“For my resit* [second sit]*, my friend gave me a load of mark schemes that she had been given from the CLIN skills stock, which I didn't know about… If I'd just had access to that when I was in my first sit… I would have known what to do.”*

Peer networks played a particularly significant role in access to learning materials, frequently serving as gateways to alternative resources which were otherwise unavailable. Students with fewer social connections, were less able to benefit from these informal systems, thereby reinforcing inequalities in first sit preparedness.

*“Group chats…that’s where most resources are being shared and distributed among peers… if* [students] *aren’t aware of these group chats… they will be missing out on a lot of resources.”*

*"The part where they* [the university] *match you with somebody else that happened to fail first year but then passed second year… was very, very helpful."*

Improved awareness and access to resources enhanced both preparedness and confidence, enabling more informed and meaningful revision as students were more focused and efficient in their preparation and shifted their study strategies. Their view was that earlier and more equitable access could potentially have improved their first-sit outcomes.

#### Financial factors

Financial constraints also shaped students’ ability to access resources and commit adequate time to examination preparation. This included both the direct costs of study materials (e.g., apps and subscription platforms) and broader financial pressures that required students to balance study with paid employment or family responsibilities.

Prior to the first sit, financial pressures limited some participants’ engagement with reportedly effective (but costly) resources and reduced the time available for structured revision. Uncertainty about which tools were genuinely beneficial compounded the challenge.

*“Not all resources that are really good and that are reliable, are free… You felt pressured, like should I buy that as well? Should I subscribe to that? Would it actually make a difference?”*

*“That was a barrier, like thinking I’ll have to pay £30 for this* [*Anki app*]*. Is it worth it? “Finances play a part, I had to work… a lot of ethnic minority students are supporting families.”*

After failure, many participants reevaluated their resource strategies. Many became more willing to make short-term financial sacrifices by reducing working hours or purchasing previously avoided materials; others relied on informal solutions such as sharing accounts, borrowing tools or accessing peer-recommended resources. These shifts prompted more strategic decision-making and behavioral changes, motivating students to focus on the resources most likely to enhance second sit preparation and engage with them more effectively in a strategic approach.

*“I had to work a lot on the side due to financial reasons. I had to rely on my overdraft at that point in my life… With a lot of sacrifice, I had to give it my all to pass this time.”*

*“It was… a lot of sacrifice financially, just to be able to focus on that gap between the 1st and 2nd sit. I know a lot of my peers resonate with this feeling.”*

Importantly, financial pressures were not tied to any single ethnic group. Participants across various backgrounds described financial strain, and experiences varied within groups. Financial challenges therefore represents a broader contextual factor that may hinder examination preparation for students. While not usually the primary determinant of success, they shaped students’ access to resources, the strategies they adopt, and their engagement with study materials, highlighting the interplay between financial, social, and structural factors in academic outcomes.

### Theme 3: support

Support was a recurring theme across all interviews, with participants reflecting on their experiences both before and after their first-sit examinations. Support encompassed academic, pastoral, and social elements and was viewed by students as a key factor influencing their overall experience and performance. Participants’ accounts highlighted clear differences in both the availability and experience of support across the assessment lifecycle. Support was reported to be often limited, unclear, or difficult to access prior to a first sit examination, but became more visible, personalised, and proactive following examination failure.

#### Institutional support

A prevalent sub-theme was the participants’ access and engagement with institutional support processes, including, but not limited to, personal tutors, formative feedback, mitigating circumstances, and structured academic interventions such as targeted teaching sessions. While all participants acknowledged that institutional support structures were available, many described challenges in navigating, engaging with, or benefiting from them prior to first sit examinations.

Participants described mixed experiences of institutional support. Although teaching staff were generally viewed as well-intentioned, interactions were often described as limited in-depth or insufficiently tailored to individual needs. Some students felt unsure how to approach staff or lacked the confidence in articulating their difficulties, which reduced meaningful engagement. Support was typically perceived as something students needed to actively seek out, rather than being proactively offered. As a result, institutional support prior to exam failure was often experienced as superficial or passive, limiting its effectiveness in preventing underperformance. However, the protective role of early and proactive institutional support as highlighted by students illustrated how institutional support can function as a preventative mechanism in students underperformance.

*“I did not do so well in my formative and was signposted to additional help, I think these are the points where people could start signposting to mentors, or tutees.”*

Participants consistently reported that institutional support improved noticeably following examination failure, becoming more visible, tailored to their individual needs, and actively offered, which enabled more meaningful engagement with both academic and pastoral guidance.

*“The most I felt supported was the 2nd sit… More one to one, more individual. Prior to that, I didn’t really… so I just suffered in silence”*

*“There’s definitely more support second time around.”*

Personal tutors emerged as particularly important, with contact shifting from minimal or infrequent meetings to regular, focused engagement, tailored to the student’s individual needs. Participants valued both the pastoral and academic dimensions of this support, though the relative importance of each varied between individuals. For some, personalised guidance clarified expectations and learning objectives, while for others, proactive staff intervention ensured that existing or previously unaddressed learning needs were better recognised and supported, enabling adjustments that had previously been overlooked or underutilised.

*“My personal tutor could tell that something was wrong and they told me to apply for mitigating circumstances and it saved my place at Med school, because otherwise I would have been kicked out.”*

*“Personal tutor I definitely spoke to. She was really helpful… really supportive and kind. She definitely tried her best to help me think of genuine steps to move forward …to prepare for my resit examination.”*

These interventions were critical in facilitating academic recovery and, in some cases, ensuring continuation at medical school. Students reported receiving clearer feedback on strengths and weaknesses, which facilitated targeted and effective revision, and increased access to staff-led small-group teaching and one-to-one feedback.

*“There was a one-to-one meeting where you have a staff go through your weaknesses. It really gave me a focus on what I should be revising more. I wish that could have been available before….”*

Participants described significant personal and cultural barriers to seeking institutional support prior to examination failure. These barriers included fears of being judged, uncertainty about the legitimacy of their difficulties, and norms encouraging independent coping. As a result, institutional support was often underutilised even when available.

*“I think it’s a stigma…of not wanting to label yourself as dyslexic or ADHD…it’s also why I think EM students don’t go for extenuating circumstances or don’t seek out help.”*

*“I almost didn’t access mitigating circumstances because I was minimising my problem… I didn’t feel it was valid for me to do so.”*

Examination failure appeared to act as a legitimising trigger for seeking support from the institution with students reporting that they felt more confident approaching staff and more willing to engage with institutional systems. Students highlighted the importance of personalised institutional support in promoting their academic success. The increased access to institutional support was associated with enhanced engagement with learning resources both working synergistically to enable more strategic and effective examination preparation. Overall, these findings underscore the importance of timely, tailored, and well-communicated institutional support in enabling students to maximise their potential.

#### Peer support

Peer support is the academic, social, and emotional assistance students gain from fellow medical students including mentorship from senior students. Prior to the first sit, participants frequently reported limited engagement with peer support, often due to lack of awareness of existing networks, social barriers, or practical constraints such as commuting or family responsibilities. Many students were unintentionally excluded from informal study groups or peer networks where others would practice together, discuss examination expectations, techniques and share study strategies.

*“What I didn’t realise, is everyone was practising OSCE’s in their friendship groups… whereas I was missing out on all of those things and didn’t realise.”*

This limited integration not only reduced opportunities for collaborative learning and strategic revision but also constrained access to peer-shared resources. Consequently, participants relied heavily on solitary study or formal lectures. These early barriers demonstrate how the absence of peer interaction could impede not only academic preparation but also self-assurance in approaching assessments.

*“Group chats—I think that should be highlighted because that’s where the resources are being distributed amongst peers. I think having insight into how others are doing in their revision, and a lot of students aren’t aware of these group chats they can access.”*

*“I commute from home and spend at least an hour each way…I was aware that I did not have friends, whereas other students were bonding a lot more outside sessions.”*

*“I don’t think it’s very easy to get support but a lot of the white students they’re able to get better peer mentoring and stuff like that because they’re able to find like-minded students in the senior years, they’re able to like be included”*

After failing the examinations, participants consistently reported increased engagement with peer support, facilitated both through informal networks (friends and study groups) and formal university mechanisms such as mentorship programmes. Peers provided critical, examination focused feedback, shared resources which participants had previously been unaware of, and supported practical preparation for clinical assessments. For many students, peer support acted as an essential academic lifeline, improving both engagement and strategic revision. Beyond the academic benefits, peer interactions offered motivation, reassurance, and accountability, helping students maintain a positive mindset and persevere during challenging second sit periods. All participants who had experienced examination failure noted that confiding in peers, friends or mentors helped them process the setback and it encouraged active participation in both peer and institutional support networks. Peer support also complemented access to learning resources, enabling students to use materials more effectively and gain clarity on examination expectations. Together, these student accounts highlighted how peer networks became a critical component boosting confidence and emotional resilience post failure.

*“I’d say two main changes* [between first sit and second sit examinations] *were probably more recall, and…a more supportive, specifically, study network.”*

*“I didn’t practise with any peers for the first sit, but for the second one I did with someone who passed first time… that really gave me confidence.”*

*“Student-led seminars and resource sharing were really beneficial… they had the insight and contextualised it…”*

“*Mentoring would be the best thing ever… someone to actually be there specifically for you… to say, ‘we understand your struggle’.”*

Engagement with peer support is closely linked to socio-cultural factors, demonstrating how early experiences of belonging and integration within the cohort can influence later academic success. Many participants reported initial isolation or limited access to networks due to logistical, social or cultural barriers restricted opportunities for collaborative study and the informal sharing of resources. This early lack of integration contributed to lower preparedness and confidence for assessments. Overall, peer support played a pivotal role not only in providing academic guidance and improving academic outcomes but also in providing motivation, reassurance, and emotional support throughout the preparation period for examination. It highlighted the important role of both formal and informal peer support in shaping academic success and sustaining student wellbeing.

### Theme 4: personal

This theme focused on the internalised experiences of students. It captured the powerful emotional responses to the high-stakes academic environment, particularly the fear of failure and a stereotype threat being a source of intense anxiety and self-doubt leading to lack of confidence before and during examination. There was also the perceived stigma by EM students associated with asking for help and support. Across accounts, stereotyping, stigma and fear shaped students’ confidence, help-seeking behavior, engagement with learning and influenced examination success.

#### Overcoming stereotype

A pervasive finding amongst participants was a deep-seated fear of failure, often described as a fear of being kicked out of medical school. For students from ethnic minority backgrounds, this fear was linked to an exaggerated sense of imposter syndrome with internalisation and conformation to the stereotype that they are not good enough to be in medical school. Several participants described how fear and anxiety undermined their performance and confidence in their own academic ability during first-sit examinations:

*“The first thing that will come to my head is the fear of getting kicked out.”*

*“I kept doubting myself a lot in the first examination papers…”*

While this fear was a source of intense anxiety, self-doubt, and overthinking during the first sit, it became a forceful primary motivator to passing the second sit examination as students from ethnic minority backgrounds reported they became more focused, more determined in their preparation and a positive shift in their mindset (e.g., “military mindset”). Many reported marked improvements in confidence during their second sit, particularly in clinical examinations, decision-making and reduction in second-guessing, which they perceived contributed to improved performance.

*“I had to overcome that stereotype doubt and really focus on the revision to really prove I’m supposed to be here.”*

*“I don’t know how you build confidence in people, but if there was a way to do that… because it’s not that we weren’t capable of passing on the first sit, when I did OSCE’s the second time around, I passed every station… I got almost full marks in most stations.”*

#### Destigmatisation

This was another significant area that participants identified. Participants described a strong personal hesitation to seeking academic, pastoral, and institutional support, such as applying for mitigating circumstances. This hesitation stemmed from a fear of seeming weak, a fear of not being believed by the institution, and a personal tendency to minimise their own difficulties as not big enough to justify support. Students described their reluctance to seek support even when aware that it existed, often anticipating rejection or negative judgement:

*“I think them (the school) not believing you… the fear of how we are perceived… or wanting to put up a brave face that I’m actually OK, I don’t need help.”*

*“EM students aren’t as vocal for help to ask for help, or they don’t think they would get the help compared to our white counterparts.”*

*“I think my white peers are more open to divulging things that happen to them.”*

Some participants described internalising their difficulties and minimising their need for help. This delayed access to academic and learning support, including identification of additional learning needs with support only sought after the first-sit failure.

“*I was under the whole ‘people have bigger issues in the world’… I was like ‘I’m fine, I’m fine, I’m fine’ and realised I wasn’t fine too late.”*

*“No one’s ever asked me, ‘have you done a dyslexia screening?’… it took the second sit … I think it’s a stigma of not wanting to label yourself.”*

Overall, fear of judgement, concerns about legitimacy, and norms around self-reliance led many students from ethnic minority backgrounds to avoid or delay accessing support prior to first sit examinations. Failure appeared to act as a legitimising trigger, reducing these barriers and enabling greater engagement with academic and institutional support during examination second sits. They approached personal tutors, disclosed difficulties, and engaged with support mechanisms including mitigating circumstances, learning support, and targeted academic interventions more readily without fear of stigmatisation, perceiving them as necessary. As a result, they felt more supported, less isolated and better equipped during their second-sit examination preparation.

*“I felt more comfortable asking the teachers, what do you actually want in the exam?”*

*“It could be the lack of reaching out for support before the first sit for us EM students… when everything they have tried on their own fails … in the second sit then they reach out for support.”*

## Discussion

This study examined the factors perceived to have contributed to EM students’ improved performance in second sit examinations and whether these could be transferred to first sit examinations. As noted in the introduction there is an attainment lag between our white and ethnic minority medical students as there was a significant differential performance in the first sit examinations which disappeared by the second sit examinations. We explored and checked to ensure there were no examination-related reasons for the improved performance and found none as both assessments were designed and delivered through the same process. This is because at Warwick Medical School there is a very high commitment to ensuring that all summative end-of-year examinations (written and Objective Structured Clinical Examinations) for both first sit and second sits are reliable, valid and meet the standards set by the Medical Schools Council Assessment Alliance (MSCAA) (20). As such, each examination prepared for first sit and second sits are inherently similar and subjected to robust quality assurance (QA) and standard setting processes that involves a collaboration between the assessment team, academics and clinical partners. The process is designed to ensure that questions used in each examination sit are high quality and rigorously test the intended learning outcomes for the stage of the learner in relation to the curriculum. This supported our findings that there were other factors other than examination related involved in this attainment lag. Examination-related factors, such as familiarity with examination formats, may contribute to performance in second sit examinations, however, at Warwick Medical School, all examinations (formative and summative examinations) throughout the Programme are conducted using the same assessment format. Students are therefore exposed to this format repeatedly during their training and are likely to be familiar with examination format structure, making this argument less likely in our context.

The result of our study illustrated the complex and multifaceted factors that led initially to failure and subsequently are perceived to improve performance in second sit examinations. Drawing from students’ personal perspectives, it offered a realistic understanding of the reasons behind initial underperformance. According to the findings of this study, EM students recognised the reasons behind examination failure and attributed these to socio-cultural factors, access to resources to aid learning, accessibility of both institutional and peer support and personal factors (which they acknowledged shaped some of their own choices). The voices of participants provided in-depth insight into the strategies and actions taken to achieve success when preparing for and undertaking second-sit examinations.

In our study, participants reported feelings of being disconnected from peers, the medical school community, and the wider university. This manifested prior to first sit examinations as feelings of isolation and exclusion that prevented them from feeling immersed within the community of learners and connected by a sense of belonging. Similar observations of not belonging have been reported by other authors (21) and the misperception of individuals making assumptions that they cannot be part of a community due to perceived differences often leading to them feeling excluded (22). Data from the study highlighted students’ perception of how not being engaged and interacting with their community of learners through social networks impacted both their social and academic performance. Our finding of the influence of social network on examination performance is similar to that reported in a longitudinal study which showed a correlation between social networks and examination results (23). In that study, they found that friendships and social relationships formed at the start of a course were more likely if students were from the same ethnic groups. In addition, if students linked to a social group were performing well then the whole group performed well and similarly if the social group were underperforming then this was reflected across the group.

These findings on social networking, access, community and belonging, could be viewed through a sociological lens and fit with the social learning theory of Communities of Practice by Wenger (24). Wenger defined Communities of Practice as groups of people who share a concern or a passion for something they do and learn how to do it better as they interact regularly. Wenger conceptualized it as a social theory of learning that views engagement in social practice as the fundamental process by which we learn and social participation being central to learning. This resonates with our findings as prior to the first sit examinations, participants in our study were unaware that students were learning and rehearsing clinical skills together in their friendship groups. It later became clear to them that their limited integration not only reduced opportunities for collaborative learning and strategic revision but also constrained access to peer-shared resources. Wenger’s description of how communities develop their practice through a variety of methods, including problem solving, requests for information, seeking the experiences of others, reusing assets, discussing developments, mapping knowledge and identifying gaps were pertinent to our findings.

Financial constraints emerged from the voice of the participants in relation to the cost of additional, specific learning materials that attracted a financial cost (e.g., apps and subscription platforms). The authors appreciate that financial pressures are not specific to any single ethnic group and that white medical students also face similar financial strain and hardship. However, our study does raise the issue of intersectionality of socioeconomic status and ethnicity. There is no doubt that a student’s financial capital (ability to buy apps for learning) and / or social capital (knowledge of which apps to buy) can affect learning, knowledge acquisition and assessment performance thereby contributing to the concept of a ‘hidden curriculum’. Those two aspects (financial and social capital) seem to be equally at play in impacting our ethnic minority student participants. However, it will be interesting to see if the findings remain the same with larger cohort of students’ groups.

Participants in this study identified accessibility of both institutional and peer support as a significant contributor to their success or failure. Before the first examination, participants acknowledged that they did not actively seek support due to a deep-seated conception that this would be interpreted as being weak, fear of discrimination and risk of attracting negative judgements from academics or peers. Support structures, revision resources, mentoring schemes, and opportunities for peer learning were consistently available, yet engagement with them often occurred only after students underperformed. Participants recounted that sometimes they had difficulty navigating support as they did not have a clear understanding of what support was available to them in preparing for examinations. Notably, strategies and support mechanisms in their view tended to surface more openly only after they had failed the examination. Following failure, participants described a clearer understanding of assessment expectations, greater awareness of effective revision tools, stronger engagement with peer networks, and more personalised interaction with institutional support structures.

Positive actions to integrate with peers, engage with a variety of formal and informal mechanisms resulted in access to resources, support, strategic revision approaches that ultimately improved examination performance according to participants. In addition, all these changes made in preparedness for second sit examinations also helped EM students develop a greater sense of belonging, effective networking, emotional resilience and improved confidence. These findings suggest that fostering a sense of belonging and early integration with the cohort of learners provides important peer support, generates rapid sharing of educational resources, reduces isolation and maximise the probability of first-time success. It also reinforces how leveraging social networks can enhance engagement and collaboration with peers and impact academic performance (25).

A novel finding in this study is that internal barriers to help-seeking, shaped by stigma, cultural expectations, and fear of judgement became reduced as failure legitimised the need for support and the need to change. Personal factors, emotional responses to the fear of failure, and the negative impact of stereotyping are similar to those reported by other authors (26). The perception of students in this study that they must work twice as hard as their white counterparts is also reflected in the literature (27). The psychological change in overcoming this stereotype threat and the destigmatisation of accessing support helped created an environment in which students were better positioned to succeed.

There was broad consensus that cultural responsibilities contributed toward examination failure. Participants perceived that their level of cultural responsibility to family is not shared by other ethnicities, and this resulted in them experiencing increased burden and self-isolation compared to peers. These responsibilities acted as barriers to becoming immersed in the community of learners and reduced their ability to integrate on a social level and establish friendship and allegiances for study opportunities. Furthermore, as graduate entry medical students the perceived burden of family responsibilities may not only be obligations to their own immediate family but to wider family members. Other qualitative studies have shown similar lived experiences of EM students when managing challenges in medical education (28).

While this study focused on ethnic minority students, we believe the mechanisms identified are not entirely unique to this group. Challenges related to peer integration, confidence, and help-seeking are likely to affect a wide range of students, particularly those with intersecting responsibilities such as caring roles, commuting, financial pressures, or additional learning needs. However, the important thing our findings have shown is that many of the factors perceived to underpin second sit success were already present prior to first sit examinations, but were not experienced by students as accessible, visible, or relevant at that stage.

## Limitations

This study is limited by the relatively small number of participants and therefore has limited generalizability. However, despite this limitation, the study generated deep insights by exploring the lived experiences of EM students on a graduate entry medical course. It provided valuable understanding of potential mechanisms underpinning and contributing to differential outcomes; the initial underperformance and subsequent improvement of EM students sitting examinations.

Another limitation is the overlap of identities within the participants group. We do acknowledge the difficulty in capturing important differences between ethnic subgroups and the distinct challenges each ethnic group may face. Due to the small numbers of participants within individual ethnic groups, analyses were conducted using an aggregated ethnic minority category. Consequently, the findings should not be interpreted as reflecting the experiences of any specific ethnic subgroup. However, by centering participants’ voices and experiences, we believed the study provided enough understanding of the cultural, social, and institutional factors and processes influencing examination outcomes in EM students in general. It also highlighted areas where further support may be needed.

Finally, as with many qualitative studies, there is a potential for self-selection bias, as students with particularly strong opinions or challenging experiences may have been more likely to participate. However, in our view, there will always be overlap between the challenges faced by the wider EM student population, hence the findings remain relevant as they can be used to inform comprehensive strategies for support and intervention that benefits all EM students.

## Conclusion

The participants in our study did not report a sudden improvement in their academic ability. Instead, they described changes in their networks, access to resources, level of participation in institutional and peer support and personal confidence. These shifts influenced how they approached assessment preparation and interacted with the medical school environment. As a result, improved performance in second sit exams was not seen as a reflection of academic capability alone, but rather as the outcome of conditions that fostered more strategic, well-supported, confident learning and preparation.

By identifying what changed between attempts, this study shifted the focus from remediation after failure toward understanding how the conditions experienced by re-sitting students might be implemented proactively before first sit examinations. This highlighted opportunities for earlier intervention, potentially reducing the need for students to encounter failure before gaining access to the tools, strategies and support required for success.

Our findings suggest a critical disconnection; the conditions necessary for success frequently became actionable only after failure. This points to issues of awareness, access and timing rather than a lack of provision and reframes failure not as an isolated academic event, but as the product of longer-term social, structural, and personal dynamics intersections. The findings suggest that EM students may experience these challenges more acutely, particularly in relation to belonging, representation, cultural expectations, and stigma around help-seeking. In this way, ethnicity appears to operate within a wider network of intersecting influences rather than as a standalone factor. Therefore, this study emphasises the need to make EM students aware of positive actions that can be taken to ensure better academic performance and the importance of being an active member of a community of learners.

### What this study adds

A key strength of this study is the translation of findings into a structured set of actionable recommendations (Box 1). These recommendations are organised into the 4 core themes reflecting the multi-dimensional nature of differential attainment identified in this research. Importantly, the recommendations move beyond high-level suggestions and instead provide specific, implementable strategies that medical schools can adopt within existing educational frameworks.

The contributions of our research project are fourfold:

- For educators and scholars, it adds to the broader literature, offering a deeper understanding of the mechanisms underpinning underperformance of EM students and by extension the awarding gap in a medical school context.
- For medical schools, the findings provide direct, actionable insights into specific areas requiring intervention, such as the need for proactive support systems and culturally competent pastoral care.
- For universities and policymakers, this research contributes to a more comprehensive understanding of educational inequality, informing strategies to promote equity in higher education.
- For society, by investigating barriers to success in undergraduate medical training, this study supports the goal of fostering a more diverse and equitable medical workforce that is better prepared to serve the needs of a diverse population.

Collectively, these recommendations provide a practical framework for addressing awarding gaps and align with broader institutional and policy priorities focused on equity, inclusion, and student success.

BOX 1 **Recommendations.**

These recommendations are derived from participants experiences and the interpretation of the study findings.

**Table:** 

Theme	Sub-theme	Recommendation
1. Socio-cultural	1.1 Cultural differences	Design engagement activities to promote networks between various groups of learners aimed at fostering a sense of community and belonging.
	1.2 Community and belonging	
	1.3 Networking	Actively promote inclusion of all cohort in social network activities, e.g., peer group meetings
2. Resources	2.1 Access to resources	Improve signposting to accessible resources including free resources and resource sharing.
		Improve visibility of key materials including:
		A dedicated resource for assessment to outline assessment expectations
		Revision tools and techniques to develop a more strategic approach
		Recorded video (talking head) explaining the assessments (written and clinical examinations)
		Reinforce the importance of formative assessments to drive learning and prepare for summative assessments
	2.2 Financial factors	Detailed signposting to university services: student services, library, student finance and additional types of financial support available.
3. Support	3.1 Institutional support	Reinforce the role of the Personal Tutor (PT) and Clinical Personal Tutor (CPT) at different points throughout the academic year.
		PTs and CPTs to have tailored tutor meetings with more emphasis on assessment at least 8 weeks prior to an examination.
	3.2 Peer support	Introduce a peer-peer buddy / mentor system and the importance of learning with and from each other
		Encourage peer group chats and small-group student led teaching
4. Personal	4.1 Overcoming stereotypes	Run staff-student sessions on cultural awareness and destigmatisation of accessing support and guidance.
	4.2 Destigmatisation	Develop student support webpages that are easily accessible, user-friendly, intuitive and interactive.

## Data Availability

The raw data supporting the conclusions of this article will be made available by the authors, without undue reservation.
